# Editorial: Application and mechanism of plant biostimulants, biochar, fertilizer products, and other nutrition-related agrochemicals

**DOI:** 10.3389/fpls.2025.1570209

**Published:** 2025-05-30

**Authors:** Meng Xu, Joao Antonangelo, Rabia Nazir, Roberto Ruggeri

**Affiliations:** ^1^ State Key Laboratory of Efficient Utilization of Arable Land in China, Chinese Academy of Agricultural Sciences, Beijing, China; ^2^ Key Laboratory of Plant Nutrition and Fertilizer, Ministry of Agriculture and Rural Affairs, Chinese Academy of Agricultural Sciences, Beijing, China; ^3^ Institute of Agricultural Resources and Regional Planning, Chinese Academy of Agricultural Sciences, Beijing, China; ^4^ Department of Crop and Soil Sciences, Washington State University, Pullman, WA, United States; ^5^ Council of Scientific & Industrial Research, Islamabad, Pakistan; ^6^ Department of Agriculture and Forest Sciences, University of Tuscia, Viterbo, Italy

**Keywords:** biochar, biofortification, biostimulants, microbial fertilizer, nano fertilizer, organic fertilizer, plant nutrition regulator, trace fertilizer

## Introduction

Plants need to be fed to produce enough grains and fruits to meet human demands. Humans have been pursuing more efficient and green agricultural inputs to meet the nutritional needs of plants, and have also been constantly updating the outdated application methods to enhance the absorption of nutrients in plants. Historically, the transformation of organic manure to chemical fertilizer as the dominant substance for plant nutrition is closely related to the Industrial Revolution. The Industrial Revolution promoted the development of the chemical industry, providing the technology and equipment needed for the mass production of chemical fertilizers. The emergence and application of chemical fertilizers have also greatly increased plant productivity, supporting global population growth and urbanization process, which in turn furthered the development of the Third and Forth Industrial Revolutions. Today, urban agriculture and vertical farms are also gradually emerging, and these new scenarios also require new nutrient management models. Meanwhile, plant biostimulants and biochar are two emerging categories of agricultural inputs related to plant nutrition. Plant biostimulants are expected to become the third major type of agricultural chemicals after chemical fertilizers and pesticides. Besides being used as a soil conditioner and for environmental remediation, biochar is also expected to become an indirect nutrient input for enhancing crop productivity. Novel materials, such as coating material, nanomaterials, metal-organic frameworks (MOFs), covalent organic frameworks (COFs), hydrogen-bonded organic frameworks (HOFs), and crystalline porous organic salts (CPOSs), have also been used to regulate chemical fertilizers for plant efficient absorption. Of course, organic fertilizers continue to be applied worldwide, and their effectiveness is usually enhanced by adding better microbial agents and combining them with chemical fertilizers and biochar (Rathinapriya et al.). Plant physiologists, microbiologists, soil chemists, and other researchers need to explore the mechanisms of action in greater depth, such as how these substances are efficiently absorbed, transported, and utilized by plants, and how they function to promote growth, enhance stress resistance, and improve yield and quality (Cai et al.). By revealing these mechanisms, agricultural chemists could further optimize the formulas and application methods of these products, which is also conducive to the development of more targeted, efficient, and sustainable products. This topic covered the application effects and action mechanisms of various agricultural inputs, such as biochar, microbial fertilizers, straw, manure, slow-release fertilizers, plant biostimulants, and nanoparticles, on different crops and under various environmental stress conditions, providing rich scientific data and theoretical support for research in related special fields.

## Topic summary and contributions

During open submission period, we noticed that the live data on views and articles for our Research Topic were consistently the highest among open submission topics. We also found that there are many Research Topics similar to ours. As of January 18, 2025, among all 1918 Research Topics featured in the journal, 28 manuscripts were included in our Topic, ranking 68th; The total number of contributing authors stand at 209, ranking 35th. The authors came from 21 countries on all continents except for Antarctica. All these showed that this Topic is indeed a global hotspot in the research field of plant sciences. The scale of peer-reviewed papers from China has ranked first globally since 2021, and the same was true for our Topic. Collected manuscripts with Chinese first authors or those involving Chinese authors accounted for about half. Of course, the number of rejected manuscripts with Chinese first authors was also the largest after the peer review process. Cross-national collaborative research was not very common. This might be primarily due to the fact that many agrochemicals are still in the stage of experimentation or serving local demands, and the hardware research conditions required for the Topic Research could be met locally. This also reflected that although our Topic was a hotspot, the relative depth of research and the level of international research have not yet reached a high standard. In terms of article types, apart from the original research articles, a review and a meta-analysis paper had also been accepted for publication. Globally, nitrogen fertilizer is of utmost importance and attracts the highest number of research papers, followed by phosphate fertilizer. In contrast, potassium fertilizer has not received as much attention, with only one article submitted on this topic. Gopinath et al. verified POLY4 (a polyhalite-based fertilizer) can be serve as an excellent and environmentally friendly alternative to provide peanut (*Arachis hypogaea*) with all their necessary potassium nutrition. Based on the specific content of each article, we summarized that 9 articles related to biochar, 7 articles related to trace fertilizers and biofortification, 5 articles related to microbial and organic fertilizers, 4 articles related to biostimulants, fertilizer enhancers, and high-efficiency fertilizers, and research in other areas was relatively scattered.

## Biochar

Biochar, as a sustainable soil amendment, can significantly enhance soil quality, carbon sequestration, and crop productivity, and alleviate the impact of abiotic stress on agriculture. Rathinapriya et al. systematically reviewed the production methods of different sources of biochar, its improvement effect on soil physical and chemical properties, and its promoting effect on growth of different crops and yield and quality performance under different abiotic stress conditions. In a sense, the other 8 articles on biochar could be considered as case studies. Under salt stress conditions, Gullap et al. found that biochar, derived from olive oil pomace, could reduce the negative effects of the environment stress on the growth of forage pea seedlings. Biochar made from Spirulina (*Arthrospira platensis*) biomass achieved a very good yield-increasing effect in rice production, as demonstrated by the study of Minello et al. In experiments with and without growing ryegrass on soil contaminated with heavy metals, Antonangelo et al. found that applying biochar enhanced soil fertility, including soil pH and nutrient availability. However, excessive (2-4%) addition of biochar was not conducive to crop growth, while a low (1%, w/w) addition of poultry litter-derived biochar increased the available phosphorus and potassium nutrients required for ryegrass growth, and produced the best root and shoot biomass. Abd El-Moaty et al. for the first time developed a nano biochar by disposing of peanut shells, which demonstrated an excellent ability to alleviate plant drought stress. In selenium -rich red paddy soil, Rong et al. investigated the transfer of selenium in the soil-plant system and the effect of grain selenium -biofortification under straw return and straw biochar conditions. The idea of converting the left of crops after harvest into nutrient resources in the form of biochar and returning them to crop production is a circular nutrient concept, but it was seldom applied in soilless cultivation. Kunnen et al. attempted to explore the effect of wheat biochar on promoting the growth of wheat in a soilless cultivation system. Hou et al. investigated the optimal combination and the influencing mechanism of biochar and organic fertilizer mixed application for increasing potato yield. Liu et al. conducted an experiment on soybeans in which biochar partially replaced organic and chemical fertilizers. All the results demonstrated the great application potential of biochar in promoting the cycling of soil original nutrients and enhancing the efficiency of nutrient inputs.

## Trace fertilizers and biofortification

It is generally believed that chelated trace fertilizers are more effective than traditional trace fertilizers. In the wheat experiment by Dhaliwal et al., the effect of foliar chelated manganese fertilizer (EDTA-Mn) was not better than that of MnCO_3_ and MnSO_4_. This might be because chelated fertilizers were originally developed due to the easy ineffectiveness of traditional fertilizers in soil, rather than for foliar application. Wang et al. found that foliar application of calcium or silicon fertilizers could effectively reduce the cracking rate of a certain type of citrus and determined the optimal application concentration. The foliar fertilizers enhanced the durability of cell wall components in the fruit peel by influencing the metabolism of cell wall substances and the antioxidant system in the peel, thereby improving the resistance to cracking. Wu et al. verified the application effect of a slow-release boron fertilizer on sugar beets. This boron fertilizer was in effective in meeting the boron nutritional requirements of sugar beets in the later stages of growth, thereby promoting crop growth and improving the use efficiency of boron. Specifically, the application of boron fertilizer increased the boron transfer coefficient, improved photosynthetic performance, and promoted the transport of the shoots to the roots, thereby increasing the yield and quality of sugar beet. Piccinelli et al. verified the effect of a novel slow-release Fe fertilizer on the regreening of Fe-deficient cucumber plants. Nano trace fertilizer has always been the most advanced field of nano fertilizer. Khanizadeh et al. compared the application effects of nano Fe_2_O_3_ and bulk Fe_2_O_3_, and provided another example of how nanoparticles could enhance plant productivity compared to traditional particles. Increasing the content of minerals such as iron, zinc and selenium in harvests through fertilization is an important measure for crop nutritional biofortification. Mrština et al. found that foliar application of selenium fertilizer significantly increased the selenium content of seeds without any impact on soybean yield.

## Microbial and organic fertilizers

Since the introduction of chemical fertilizers, researchers have continuously explored how to more effectively and sustainably integrate organic materials and chemical fertilizers. Zhang et al. evaluated the effects and contributing factors of five fertilization types, including organic fertilizer application and combined application of mineral fertilizers and manure, on the yield and nutritional value of alfalfa (*Medicago sativa* L.) across China through a meta-analysis, which was crucial for optimizing the field nutrient management. The article of Yang et al. was also about alfalfa, but it focuses on the changes in soil phosphorus form resulting from the combined application of nitrogen and phosphorus fertilizers. The study of Chen et al. investigated the effects of partially substituting chemical fertilizers with straw, pig manure, and biogas residue on the yields and greenhouse gas emissions in wheat-maize rotation systems, and found that pig manure had the best effect, as it could not only increase grain yields but also reduce N_2_O emissions. New agricultural waste is also being used as organic fertilizer for field nutrient management. Chepkorir et al. studied an insect frass fertilizer made from black soldier fly frass, which performed better than other fertilizers, including commercial organic fertilizer, bio-fertilizer and mineral fertilizer, on bush bean in Africa. Several articles on biochar combined with organic fertilizer and biochar combined with organic fertilizer and chemical fertilizer have also been included in the Section of Biochar. Organic acids secreted from roots can recruit and colonize plant growth-promoting rhizobacteria to promote plant productivity. Xu et al. mimicked this natural process by using anaerobic hydrolysis of agricultural wastes (mushroom residue and tobacco waste) to produce organic acids as enhancers, which improved the application effect of plant growth-promoting rhizobacteria on maize.

Like most plants, medicinal plants cannot maintain their yield and medicinal quality when continuously cropped. Qiu et al. examined the effects of three microbial fertilizers on alleviating the continuous cropping obstacle. Among them, the complex microbial agent showed greater promise in the cultivation of *Glycyrrhiza uralensis* Fisch compared to *Bacillus amyloliquefaciens* and *Bacillus subtilis*. Interestingly, these microbial fertilizers were more effective in optimizing the structure of soil microbial communities under continuous cropping patterns than under rotation patterns. In the future, the mutual relationships between exogenous microorganisms, soil microorganisms, soil physical and chemical properties and plant growth, yield and quality deserves further exploration. Alrajeh and Sherif found *Enterobacter cloacae* promoted the growth and yield of *Curcuma longa* L. and simultaneously enhances its medicinal quality.

## Biostimulants, nutrient enhancers and high-efficiency fertilizers

Biostimulants are very popular for agrochemical companies in the real market. Our original focus was on the impact of biostimulants on yield and quality through direct or indirect effects on nutrients in the field. Farruggia et al. analyzed the effects of three biostimulants on a medicinal and aromatic plant, namely sage (*Salvia officinalis* L.), and found that fulvic acids and protein hydrolysates had a greater impact on increasing yield than seaweeds, while the influence on commercial quality was complex. The combination of biostimulants and fertilizers is becoming a trend. As we mentioned in the Background, the Value-added fertilizers, produced by incorporating bioactive substances into conventional fertilizers, are on the rise in China. Although no corresponding articles had been included, it is already very common in the global market to add substances such as biostimulants before the formation of fertilizer granules to enhance their effectiveness. This also indicates that there might be a certain disconnect between the laboratory research understood by scientists and the product demands of the market as conducted by entrepreneurs. Both non-microbial and microbial biostimulants added to low-content phosphate fertilizers tested could increase the soil available phosphorus content, but Herrmann et al. did not find that it would have a more positive effect on maize growth. Other microbial preparations and their metabolic products were classified under microbial fertilizers. Fertilizer inhibitors have also been used to control the release of nutrients. Wang and Cai investigated the effects of four major nitrogen fertilizers combined with inhibitors and oxalic acid under soil incubation condition, and results showed that the changes in different forms of mineral nitrogen under different combinations were somewhat different, but the four nitrogen fertilizers containing DMPP increased the soil NO_3_^–^N content. In low available phosphorus soil, Kurdestani et al. found that nitrification inhibitors such as Dimethyl Pyrazole Phosphate (DMPP) and Dimethyl Pyrazole Fulvic Acid (DMPFA) significantly improved phosphorus use efficiency in maize production. It has been many years since DMPP was first created and it is still under research, which indicates that DMPP is indeed a revolutionary product with long-lasting vitality. This was similar to the status of EDTA in the field of trace fertilizers.

## Conclusions

The future of plant nutrient and nutrient regulator is shaped by innovation at present. All 28 articles covered both performance studies in traditional field-based effects and matching studies for emerging demands, such as vertical agriculture and biofortified foods. The types of input products related to plant nutrition are becoming increasingly diverse, and combined applications are getting more and more complex. We believe that this Research Topic has partly bridged the gap between the potential advancements of both listed and unlisted nutrition inputs. It has also furthered our understanding of the interaction mechanisms between plants and these inputs. However, we know that although many of the novel materials reported show good performance, products that have not been tested by the market are immature. It can be much harder for a product to go from field validation to marketing than it is to go from laboratory development to field validation. This requires more funds, resources and the joint participation of experts from all aspects. On the other hand, the future research on the mechanism of input products in plant nutrition mainly involves the following three frontier fields. Firstly, more focus should be placed on researching the correlation between soil microorganism changes and the effectiveness of input products. By deeply analyzing the structure and function of soil microbial communities and their interactions with input products, scientists should aim to uncover the key mechanisms through which microorganisms enhance the transformation and release of nutrients in input products. Secondly, the impact of the inputs on plant gene expression and physiological metabolism at the molecular level should be deeply studied. By using advanced molecular biology techniques such as gene editing, transcriptomics, and proteomics, scientists should explore how inputs regulate the expression patterns of plant genes, thereby influencing key physiological processes of plants, including photosynthesis, respiration, nutrient absorption and transport, as well as the response mechanisms to abiotic stress. This research aims to lay the foundation for developing targeted and efficient inputs, achieving the goal of precisely regulating plant growth and development and enhancing crop yield and quality. Lastly, scientists should focus on the chemical characterization of the inputs themselves. By conducting a more in-depth analysis of their chemical composition, structure, and properties, as well as understanding their behavior and transformation rules within the soil-plant system, scientists should closely integrate this chemical characterization with the first two aspects. This integration helps human explore the correlation between soil microbial activity and plant molecular responses, allowing us to gain a more comprehensive understanding of the internal mechanisms of input effects. Ultimately, this will provide more precise scientific guidance for developing the next generation of inputs that are more efficient, environmentally friendly, and sustainable. If possible, we will release a second related Topic, and we welcome contributions from everyone.

